# Determination of *Aspergillus* pathogens in agricultural products by a specific nanobody-polyclonal antibody sandwich ELISA

**DOI:** 10.1038/s41598-017-04195-6

**Published:** 2017-06-28

**Authors:** Ting Wang, Peiwu Li, Qi Zhang, Wen Zhang, Zhaowei Zhang, Tong Wang, Ting He

**Affiliations:** 1 Oil Crops Research Institute of the Chinese Academy of Agricultural Sciences, Wuhan, 430062 People’s Republic of China; 2 Key Laboratory of Biology and Genetic Improvement of Oil Crops, Ministry of Agriculture, Wuhan, 430062 People’s Republic of China; 3 Key Laboratory of Detection for Mycotoxins, Ministry of Agriculture, Wuhan, 430062 People’s Republic of China; 4 Laboratory of Risk Assessment for Oilseeds Products, Wuhan, Ministry of Agriculture, Wuhan, 430062 People’s Republic of China; 5 Quality Inspection and Test Center for Oilseeds Products, Ministry of Agriculture, Wuhan, 430062 People’s Republic of China

## Abstract

*Aspergillus* and its poisonous mycotoxins are distributed worldwide throughout the environment and are of particular interest in agriculture and food safety. In order to develop a specific method for rapid detection of *Aspergillus flavus* to forecast diseases and control aflatoxins, a nanobody, PO8-VHH, highly reactive to *A. flavus* was isolated from an immunized alpaca nanobody library by phage display. The nanobody was verified to bind to the components of extracellular and intracellular antigen from both *A. flavus* and *A. parasiticus*. To construct a sandwich format immunoassay, polyclonal antibodies against *Aspergillus* were raised with rabbits. Finally, a highly selective nanobody-polyclonal antibody sandwich enzyme-linked immunosorbent assay was optimized and developed. The results revealed that the detection limits of the two fungi were as low as 1 μg mL^−1^, and that it is able to detect fungal concentrations below to 2 μg mg^−1^ of peanut and maize grains in both artificially and naturally contaminated samples. Therefore, we here provided a rapid and simple method for monitoring *Aspergillus* spp. contamination in agricultural products.

## Introduction

*Aspergillus flavus* and *A. parasiticus* are well known producers of aflatoxins, and frequently contaminate agricultural products such as peanut, maize, rice and derived products. The toxins produced by these fungi can cause considerable health risks and significant economic losses due to fungal deterioration of the agricultural commodities^1, 2^. It is therefore extremely important to detect and prevent the contamination by *Aspergillus* species or reduce the level of aflatoxins in grains used in many agricultural products.

Conventional methods for detection of *Aspergillus* fungi usually rely on plate counting that is laborious and time consuming, moreover, the number of conidia may not reflect actual damage or potential mycotoxin production because aflatoxins are produced by mycelia. Therefore, a better alternative would be required to detect the aflatoxin producers in their early stages of growth before they can produce aflatoxins.

The detection of antigens produced by fungi has enabled the development of simple, rapid, sensitivity and robust detection of specific fungi by using immunological methods^3–6^. Notermans^7^ showed that detecting mould antigen with ELISA is more reliable, specific, sensitive, simpler to perform, and is able to be used to analysis large number of samples than counting conidia for estimating moulds. And sandwich ELISA has especially advantageous: better capture of antigens, not susceptible to impurities in the sample, and can obtain reliable quantitative relationships^8^. For the past 30 years, immunoassays have been developed for detection of *Aspergillus* contamination by using polyclonal antisera^3, 7, 9^, monoclonal antibodies^10, 11^ or single-chain variable fragment (scFv) antibodies^12–14^. For *A. flavus* pathogens, however, only monoclonal antibody have been used, with a detection limit in PBS of 1~2 μg mL^−1^ by ELISA^10^ and 1 μg g^−1^ in maize or peanut by a developed scFv antibody fused to AP^14^.

In 1993, a group of Belgian scientists found a type of antibody in the blood of camelids (camels, llamas, and alpacas) produce a unique subclass of antibodies that naturally lack light chains, referred to as heavy chain antibody^15^. The variable domain (VH) of such heavy-chain antibodies is formed by only one variable domain (VHH), which contains the antigen binding site^16^. Recombinant expression of these VHHs yields a single domain heavy-chain antibody, termed “nanobody”^17^.

Unlike polyclonal and monoclonal antibodies, nanobody can be isolated together with their coding sequence by phage display, expressed with a high yield with a bacterial expression system and readily extracted from the periplasm space while still retaining their monoclonal properties^17–19^. In addition, the low expression yield and poor stability of scFv limit their development^20, 21^, while nanobodies have the advantages of strong stability, good solubility, antigen combined with good performance and low immunogenicity than scFv^22^. With these benefits, recent success in generating camelid nanobodies prompted our interest in developed and applied for diagnostic and therapeutic purposes^23, 24^, and nanobodies are promising reagents in the next generation of immunoassays.

An increasing number of nanobodies, especially in our laboratory, have been isolated against aflatoxin and applied in immunoassay^25, 26^. However, as yet not nanobody which have been described against the antigens of *A. flavus* or *A. parasiticus* have been developed into a specific and sensitive sandwich ELISA, which is able to detect the presence of these fungal species. Based on the facts, we prepared two antigens, extracellular antigens and intracellular antigens (mycelia lysate) of *A. flavus*, as immunogens to generate nanobodies and rabbit polyclonal antibody, which can recognize certain components of *A. flavus*. After panning, high-affinity *Aspergillus*-specific nanobodies, EA3-VHH, PO6-VHH and PO8-VHH were successfully isolated from an immunized alpaca nanobody library by phage display. By comparison, PO8-VHH, highly selective to *A. flavus* was finally chosen to develop a sensitive direct sandwich immunoassay for aflatoxigenic *Aspergillus* pathogens. Immunoblot analyses demonstrated the binding of the PO8-VHH to the components of extracellular and intracellular antigen from both *A. flavus* and *A. parasiticus*. Direct sandwich ELISAs based on the PO8-VHH (capture antibody) and a polyclonal antibody (detection antibody) were developed. The assay is sensitive, accurate, and cost-effective, with limits of detection as low as 1 μg mL^−1^ in PBS and 2 μg mg^−1^ mycelia in peanut or maize, making it suitable for rapid detection of *Aspergillus* spp. contamination in agricultural products. To the best of our knowledge, this is the first report of the development of a nanobody for direct and species-specific detection of *A. flavus*.

## Results

### Antigen preparation and nanobody library

To generate antibodies against aflatoxigenic *A. flavus*, extracellular antigens and intracellular antigens (mycelia lysate) from a representative strain *A. flavus* 3.4408 producing high level of aflatoxin were prepared as antigen for immunization of alpaca and rabbit. This strain was identified morphologically and chemically to be an aflatoxin producer as determined by HPLC (data not shown). After the sixth immunization, polyclonal antisera from the immunized alpaca and rabbit antisera were analyzed by indirect ELISA. The results showed a clear robust humoral response up to 1:128000. Total RNA was extracted from immunized alpaca’s blood and used to transcribe into cDNA for construction of nanobody library. A phagemid library with a size of 7 × 10^8^ clones was constructed and 20 clones randomly selected were sequenced to evaluate library diversity, which displayed a good diversity and all contained the expected inserts (data not shown) and was subsequently used for panning.

### Biopanning and phage ELISA analysis

The constructed phage library containing approximately 10^12^ phage particles was screened against extracellular antigens and mycelia lysate of *A. flavus* through three rounds of panning. In order to select phage-displayed VHHs that specifically recognize antigens, washing with PBST was increased each round by five times (10, 15, and 20 times in the first, second and third rounds, respectively), while the amount of antigens coated in plate were fixed. Under such stringent conditions, titer of the output phage was increased steadily after each round of panning, which means an efficient enrichment of specific phage (Table 1). Subsequently, the finally panned library served as a valuable pool for further selection of highly reactive antibodies.

**Table 1:** Phages applied and eluted in each round of panning by phage display for extracellular antigens and mycelia lysate.

Antigen	Round	Input(pfu)	Output(pfu)	Ratio(%)
extracellular antigen	1	6.0 × 10^12^	6.0 × 10^7^	1.0 × 10^−5^
	2	1.4 × 10^13^	1.6 × 10^8^	1.2 × 10^−5^
	3	4.0 × 10^12^	6.4 × 10^8^	1.6 × 10^−4^
mycelia lysate	1	6.0 × 10^12^	7.0 × 10^7^	1.2 × 10^−5^
	2	8.0 × 10^12^	1.6 × 10^8^	2.0 × 10^−5^
	3	3.0 × 10^12^	6.0 × 10^8^	2.0 × 10^−4^

pfu, plaque-forming unit.

**Figure 1:**
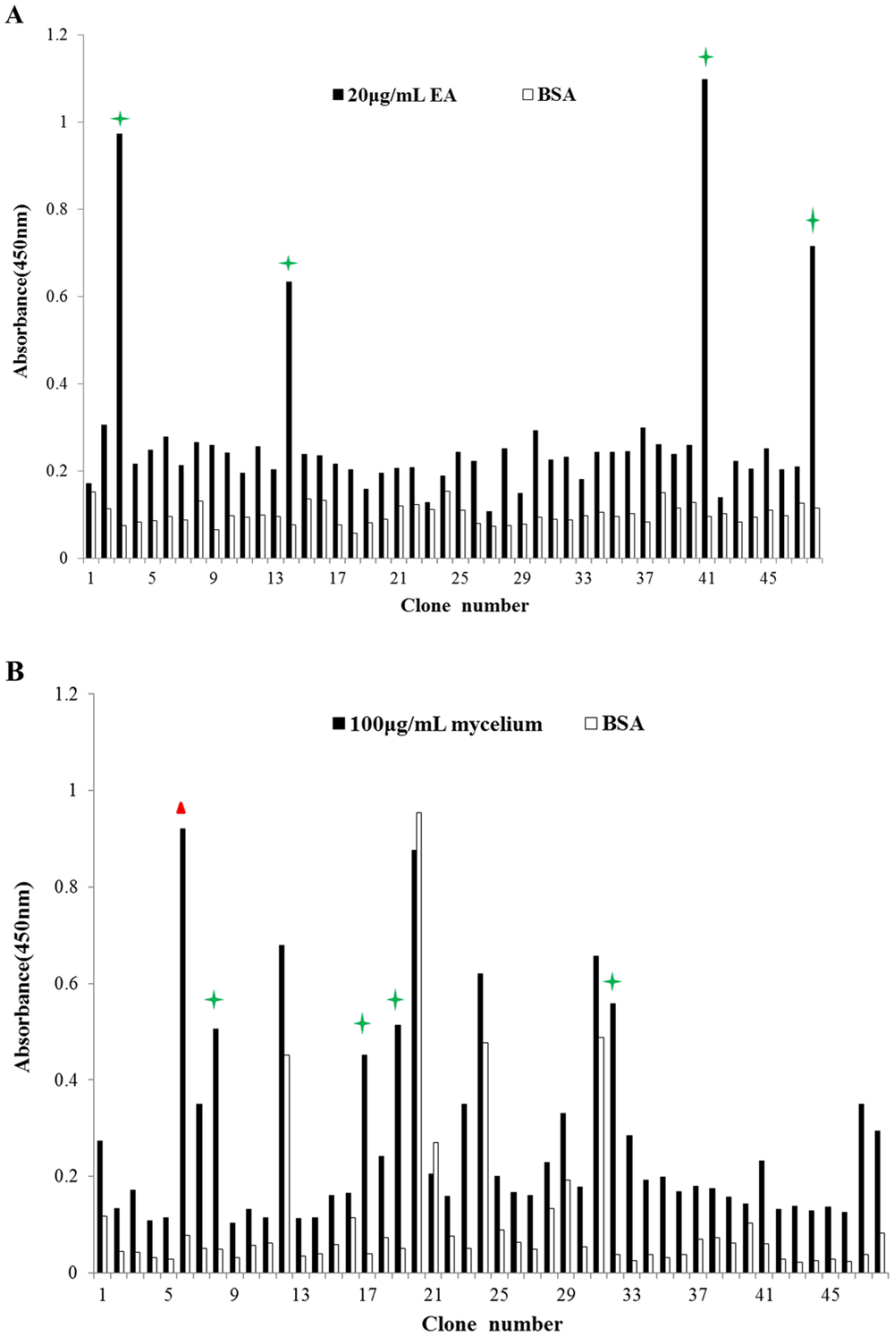
Phage ELISA of randomly selected individual clones toward extracellular antigens (**A**) and mycelia lysate (**B**) of *A. flavus*. Forty-eight clones were selected from the third round of panning and 4 clones showed positive reaction toward extracellular antigens and 5 clones toward mycelia lysate of *A. flavus* with varied signal intensity.

**Figure 2:**
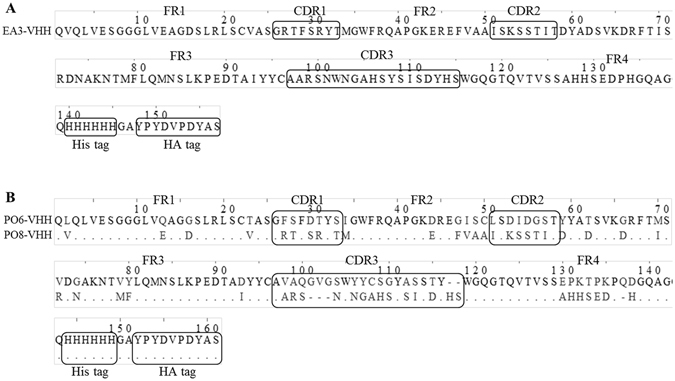
Alignment of amino acid sequences of EA3-VHH (**A**), PO6-VHH, PO8-VHH (**B**). Dots indicate identity to the top sequence and dashes indicate absence of amino acid residues compared to the longest sequence.

In order to isolate soluble nanobodies with high affinity, 48 clones were randomly selected and affinity of phage-VHH toward antigens was tested by a phage ELISA. Among them, 4 clones that exhibited a positive reaction toward extracellular antigens and 5 clones toward mycelia lysate of *A. flavus* with varied signal intensity were selected for further analyses (Fig. 1). Sequencing analyses of the selected 4 clones against extracellular antigens from *A. flavus* showed one group of antibodies: clone 3, 14, 41 and 48 carried identical nucleotide sequences (marked in Fig. 1A). These clones were then named EA3-VHH, and its deduced amino acid sequences were illustrated in Fig. 2A. And sequencing analyses of the selected 5 clones toward mycelia lysate of *A. flavus* were divided into two groups of antibodies: clone 8, 17, 19 and 32 contained identical sequences; clone 6 carried different nucleotide sequence (marked in Fig. 1B). These clones were then named PO6-VHH and PO8-VHH, respectively, and their deduced amino acid sequences were illustrated in Fig. 2B. However, further sequence analysis of these three nanobodies showed that EA3-VHH and PO8-VHH carried identical nucleotide sequences. Therefore, the phage-displayed PO6-VHH and PO8-VHH were confirmed to be originated from alpaca and were used for further characterization.

### Soluble nanobody ELISA and immunoblot analysis

**Figure 3:**
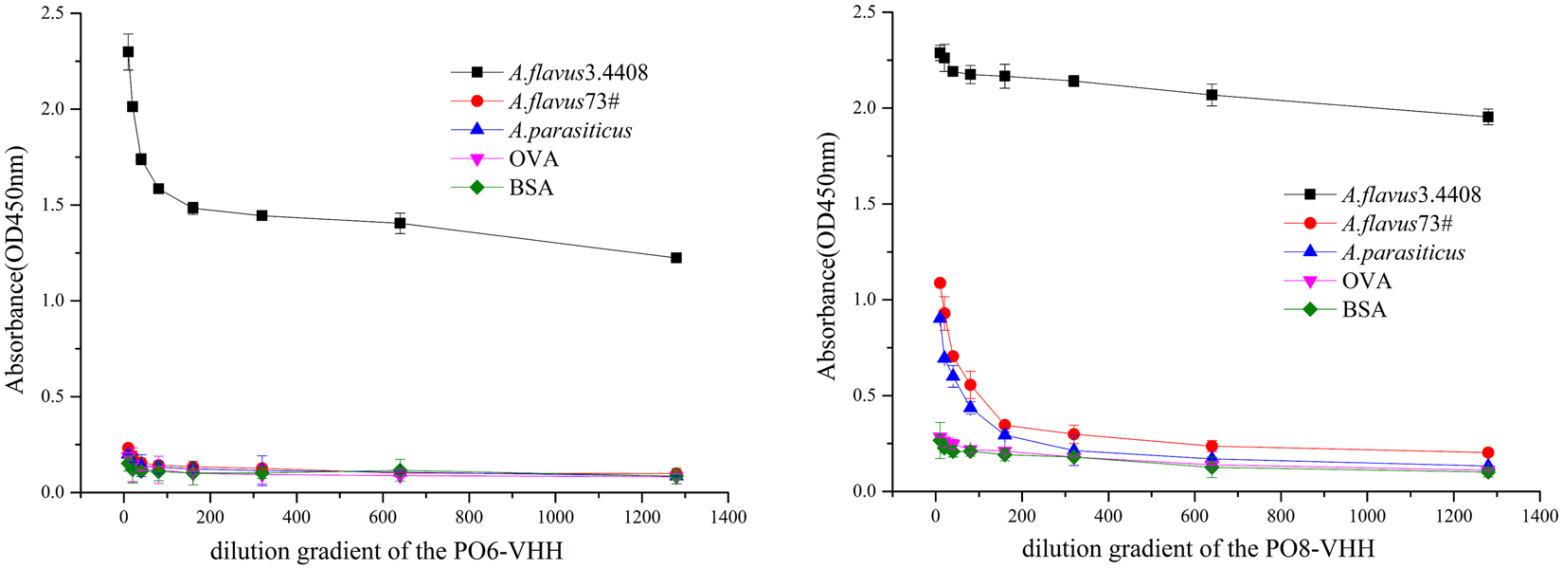
Soluble ELISAs with purified PO6-VHH and PO8-VHH.

To further reveal their binding activity toward *A. flavus* and *A. parasiticus* that also produced aflatoxins (data not shown), soluble ELISAs with two nanobodies purified from large-scale expression were carried out. The results confirmed that both still had the highest reactivity to *A. flavus* 3.4408, whereas the PO6-VHH had lowest reactive to *A. flavus*73# and *A. parasiticus* (Fig. 3). Meanwhile, PO8-VHH has a better binding ability to *A. flavus*73# and *A. parasiticus* than PO6-VHH. These results suggested that CDRs in the PO8-VHH, with variation in both the sequence and length, may play an important role in its interaction with antigens resulting in the highest binding activity.

**Figure 4:**
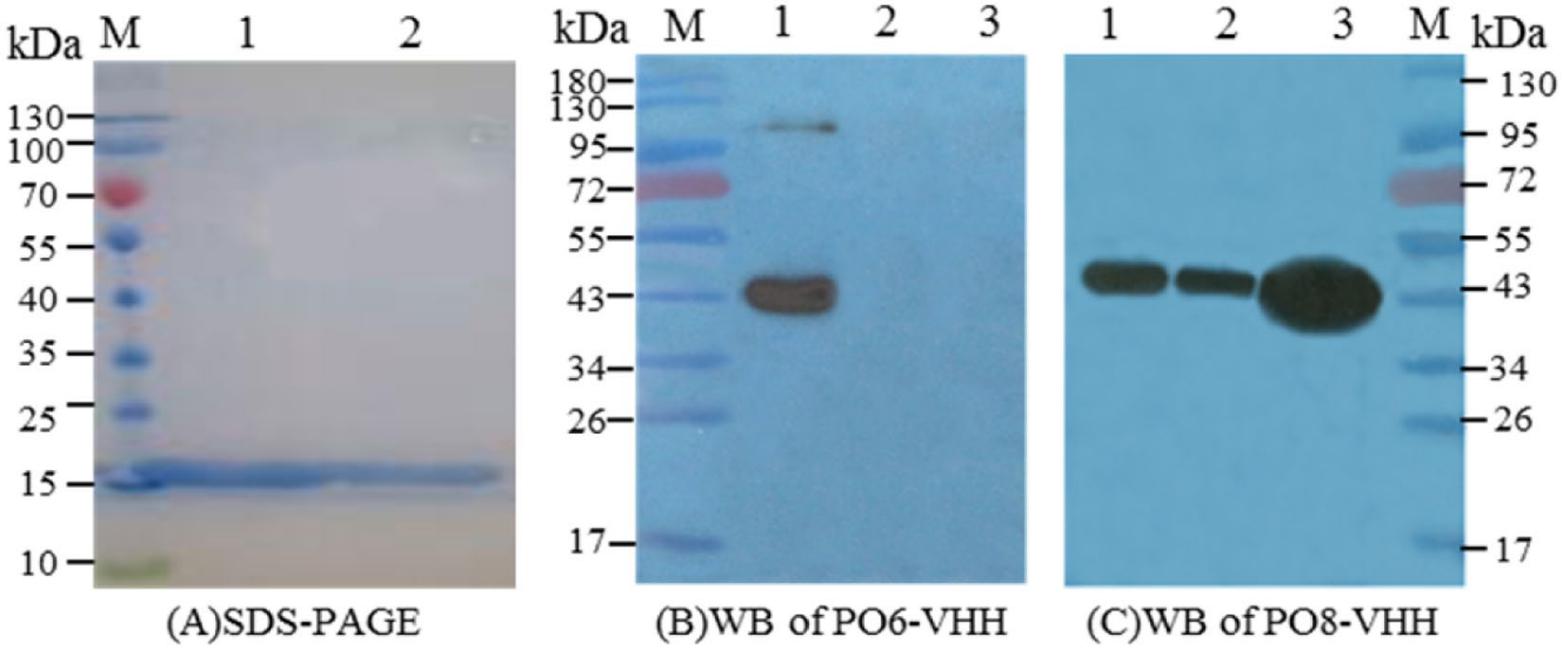
SDS-PAGE and Western blotting analysis. (**A**) SDS-PAGE profile of the purified PO6-VHH (Lane 1) and the purified PO8-VHH (Lane 2). M: Protein Marker. (**B**) WB profiles of PO6-VHH. 1: *A. flavus*3.4408; 2: *A. flavus*73#; 3: *A. parasiticus*; M: Protein Marker. (**C**) WB profiles of PO8-VHH.1: *A. flavus*73#; 2: *A. parasiticus*; 3: *A. flavus*3.4408; M: Protein Marker.

For further analyses of antigen-binding properties of the PO6-VHH and PO8-VHH, the mycelia from other *A. flavus* and *A. parasiticus* isolated from diseased peanut in China were detected by immunoblots, and the results showed that PO8-VHH could bind to the same antigen displayed a single band (45-kDa) in *A. flavus* and *A. parasiticus*, whereas the PO6-VHH could only bind to the antigen in *A. flavus* 3.4408 but not bind to other *A. flavus* and *A. parasiticus* (Fig. 4). These results further indicated that the PO8-VHH apparently contributes a proper structural conformation and is most specific to the antigen that is commonly and constitutively present on fungal mycelia of both *A. flavus* and *A. parasiticus*, although the precise component recognized by the nanobody is unknown. Therefore, the PO8-VHH may be a proper candidate for further genetic manipulation for wide use in immunoassays.

To further evaluate the specificity of the PO8-VHH, ten isolates of *Aspergillus* with different levels aflatoxin and other fungal or bacteria species (Table 2) were used for indirect ELISA analysis. The results indicated that the PO8-VHH had a high affinity for *Aspergillus*, especially for *A. flavus* and *A. parasiticus* but did not cross-react with other fungal or bacteria species (Table 2). These results congruously suggested that the PO8-VHH is specific to *Aspergillus* genus and can be able to specifically detect *Aspergillus* in the condition of complex pollution of different fungal pathogens, as Tsai pointed out that antibodies against moulds are specific at the genus level and only react to closely related genera^3^.

**Table 2:** Affinity and specificity of PO8-VHH determined by ELISA analyses.

Species	PO8-VHH for different fungi^a^	Aflatoxin B_1_(μg L^−1^)^b^
*A. flavus*3.4408	2.608	186.49
*A. flavus*73#	2.088	139.94
*A. flavus*271#	1.870	115.76
*A. flavus*233#	2.345	38.18
*A. flavus*321#	2.149	—
*A. flavus*3.2572	1.828	—
*A. parasiticus*	2.028	193.43
*A. versicolor*	0.757	—
*A. niger*	0.168	—
*A. fumigatus*	0.145	—
*Fusarium*-1	0.093	—
*Fusarium*-2	0.099	—
*Fusarium oxysporum*	0.086	—
*Penicillum citrinum*	0.080	—
*Penicillum chrysogenum*	0.079	—
*Rhizoctonia*-1	0.075	—
*Rhizoctonia*-2	0.078	—
*Trichoderma*	0.085	—
*Yeast*	0.086	—
*Escherichia coli*	0.072	—

^a^Plate wells were coated with mycelia of different fungal or bacteria species, followed by adding PO8-VHH. For wells containing the PO8-VHH, mouse anti-HA antibody and HRP-labeled goat anti-mouse antibody were additionally added. Colorimetric reactions were performed by adding TMB substrate and measured the absorbance at 450 nm. Each sample was carried out in triplicate and the scale presents an arbitrary set of the mean of OD_450nm_ readings. ^b^Aflatoxins B_1_ were determined by HPLC. *Aspergillus* and other fungal or bacteria species were cultured in Czapek medium at 28 °C for 5 days.

**Figure 5:**
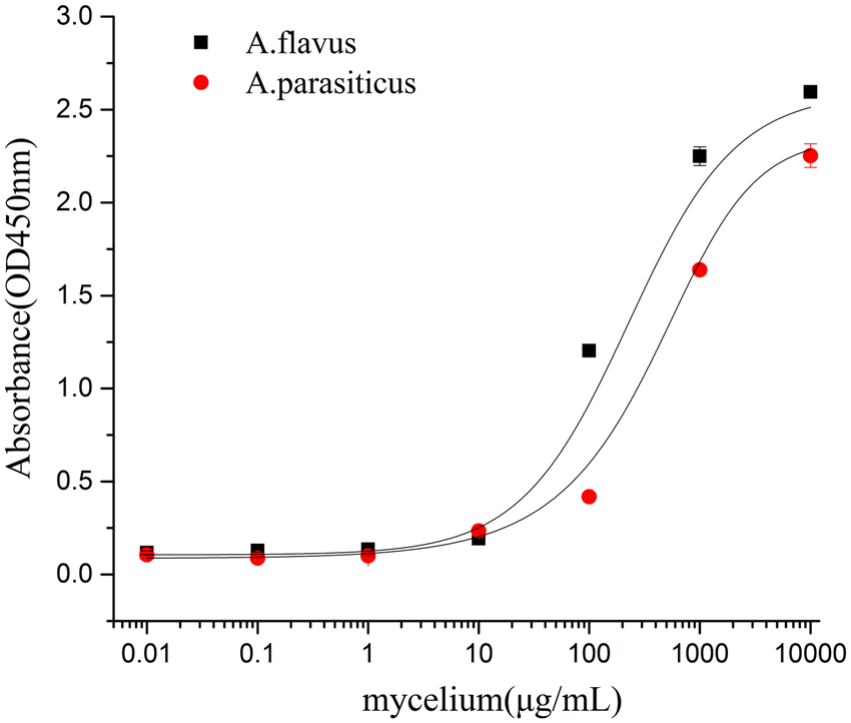
The sensitivity and detection limits of PO8-VHH determined with different concentrations of *A. flavus* and *A. parasiticus*. 100 μL of *A. flavus* or *A. parasiticus* was grinded and diluted in PBS at indicated concentrations and added into plate wells for indirect ELISA detection with 200 nM purified soluble PO8-VHH. Values represent mean ± SD of triplicate assays.

To evaluate the affinity of the PO8-VHH, the detection limit of the PO8-VHH (200 nM≈4 μg mL^−1^) was measured by indirect ELISA with different fungal concentrations. As shown in Fig. 5, the detection limit of PO8-VHH towards *A. flavus* and *A. parasiticus* was about 10 μg mL^−1^. Therefore, the PO8-VHH expressed in *E. coli* can be better used for detecting aflatoxigenic *Aspergillus* pathogens directly and simply.

### Standard curve of the sandwich ELISA

**Figure 6:**
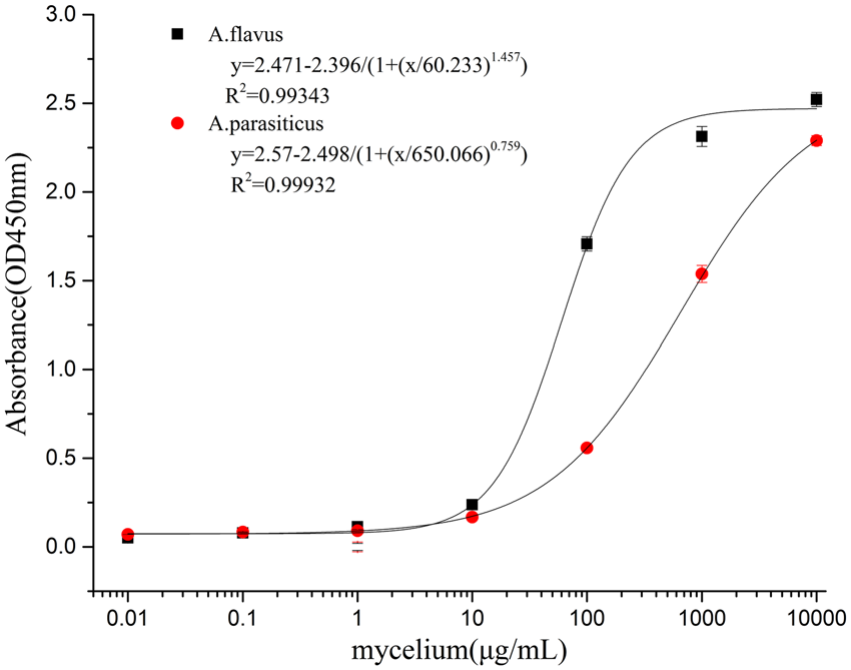
The sensitivity and detection limits of PO8-VHH as capture antibody and polyclonal antibody as detection antibody in sandwich ELISA with different concentrations of *A. flavus* and *A. parasiticus*. 100 μL of PO8-VHH (0.5 μg mL^−1^) was coated into the plate wells, followed by addition of 100 μL of *A. flavus* or *A. parasiticus* that was grinded and diluted in PBS at indicated concentrations, and then 100 μL of polyclonal antibody (1 μg mL^−1^) was added for detection. Values represent mean ± SD of triplicate assays.

To optimize sandwich ELISA detection through the PO8-VHH and polyclonal antibody, the capture antibody and detection antibody were compared for their applicability and sensitivity. The *A. flavus* and *A. parasiticus* mycelia with different concentrations in PBS (0, 10^−2^, 10^−1^, 1, 10, 10^2^, 10^3^, 10^4^ μg mL^−1^) were used to construct a standard curve. Additionally, a standard curve for the polyclonal antibody (capture antibody) and PO8-VHH (detection antibody) was also constructed, but the value of blank was so high no matter how optimized the concentration (data not shown). Finally, a standard curve for the PO8-VHH (capture antibody) at 0.5 μg mL^−1^ combined with polyclonal antibody (detection antibody) at 1 μg mL^−1^ was constructed though optimization (Fig. 6). The results showed that the detection limits were about 1 μg mL^−1^ of *A. flavus* and *A. parasiticus* for sandwich ELISA.

### Sandwich ELISA for rapid immunological detection of *Aspergillus* contamination in agricutural products

To determine the limit of quantification, different concentrations of mycelia from *A. flavus* 3.4408 and *A. parasiticus* mixed with grinded peanut or maize (1 mg mL^−1^) were prepared, and the homogenates were assayed by the optimized sandwich ELISA.

**Figure 7:**
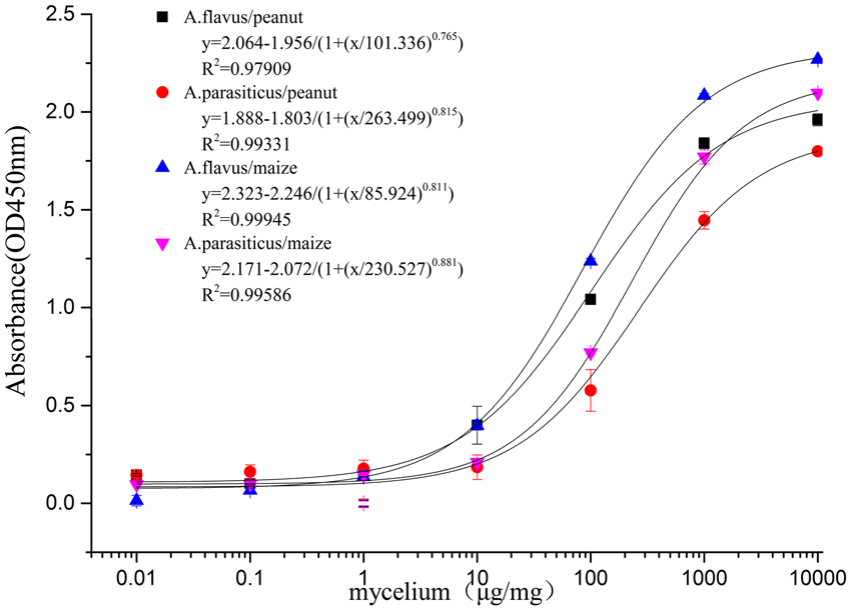
The limits of quantifications of *A. flavus* and *A. parasiticu*s in peanut and maize determined by sandwich ELISA. 100 μL of PO8-VHH was coated into plate wells, followed by addition of 100 μL mixtures of *A. flavus*-peanut, *A. parasiticus*-peanut, *A. flavus*-maize, and *A. parasiticus*-maize at indicated concentrations. Then 100 μL of polyclonal antibody was added for detection. Values represent mean ± SD of triplicate assays.

The results showed that the detection limit for the PO8-VHH and polyclonal antibody was approximately 2 μg mg^−1^, which was identical to the detection limit in PBS but with a lower and slower color development (Fig. 7). Under the optimized sandwich ELISA condition, the fungal concentration displayed a good correlation between logarithmic concentration of mycelia and OD_450nm_ values (R^2^ > 0.99 for both fungi).

To study the feasibility of PO8-VHH combined with polyclonal antibody for rapid immunological detection, sandwich ELISA was used to detect naturally *Aspergillus* contaminated peanut and maize samples collected from different areas in China and 4 artificially samples inoculated with *A. flavus* and *A. parasiticus*, respectively (Table 3). The results indicated that both samples can be detected effectively without any false-positive signals in the healthy peanut and maize. Therefore, sandwich ELISA based on the prokaryote-expressed PO8-VHH may be preferable for monitoring naturally *Aspergillus*-contaminated samples in agricultural products.

**Table 3:** *Aspergillus*-contaminated peanut and maize samples collected from different areas in China and detected with sandwich ELISA and HPLC after sample preparations.

Sample	ELISA for *Aspergillus* pathogens (absorbance at 450 nm)	Concentration of aflatoxin B_1_(μg kg^−1^)^f^
CK-peanut^a^	0.109	—
CK-maize^a^	0.086	—
AF-peanut^b^	0.517	1767.274
AF-maize^b^	0.712	1639.935
AP-peanut^c^	0.678	1449.408
AP-maize^c^	0.619	1513.999
JX31^d^	0.346	826.128
JX32^d^	0.241	143.289
FJ54^d^	0.447	1016.201
FJ56^d^	0.382	915.221
GX73^d^	0.169	44.729
GX74^d^	0.344	1088.759
GX78^d^	0.434	1079.568
HLJ98^d^	0.353	1026.486
HB106^d^	0.410	761.764
GD110^d^	0.259	326.638
GX49^e^	0.233	1060.888
GX51^e^	0.504	1226.094
GX54^e^	0.179	641.554
GX59^e^	0.329	931.604
GX259^e^	0.442	787.524
GX341^e^	0.279	830.445

^a^Uncontaminated peanut and maize. ^b^Peanut and maize were inoculated with *A. flavus*3.4408 used in this study and cultured at 28 °C for 10 days. ^c^Peanut and maize were inoculated with *A. parasiticus* used in this study and cultured at 28 °Cfor 10 days. ^d^ *Aspergillus*-infected peanut from stocks collected from different provinces, China. ^e^ *Aspergillus*-infected maize from stocks collected from Guangxi(GX) province, China. ^f^Aflatoxins B_1_ were determined by HPLC. Each sample was carried out in triplicate and the scale presents an arbitrary set of the mean of OD_450nm_ readings.

## Discussion

In this study, two kinds of antigens, extracellular antigens and intracellular antigens (mycelia lysate) from *A. flavus* were designed to include the surface proteins and crude extract proteins of *A. flavus*. In order to obtain high affinity nanobodies for *A. flavus*, we first explored the dose of antigens immunized rabbits to get high titer alpaca antiserum. Results indicate that about 1 mg mL^−1^ extracellular antigens and 100 mg mL^−1^ mycelia lysate (fungal concentration) were confirmed as good immunogen for eliciting antibodies.

In order to select phage-displayed VHHs that specifically recognize antigens, a optimized solid phase protocol was performed to select the nanobodies through 3 rounds of panning. Washing with PBST was increased each round by five times, while the amount of antigens coated in plate were fixed so as to efficient enrichment of selective phage. Finally, EA3-VHH, PO6-VHH and PO8-VHH were successfully isolated and cloned the gene of a high-affinity *Aspergillus*-specific nanobody from an immunized alpaca antibody library by phage display. By comparison, EA3-VHH and PO8-VHH carried identical nucleotide sequences. It is speculated that PO8-VHH recognized protein of *A. flavus* could be easily secreted and therefore easily detected. Therefore, it is possible to detect *A. flavus* in the samples, and this nanobody could be used to establish an immunosensor assay for detection of the samples. And results of both soluble nanobody ELISA and sandwich ELISA showed that PO8-VHH could recognizes an antigen that both exist in extracellular and intracellular of *A. flavus* and *A. parasiticus*. Specificity and reactivity assays indicated that the PO8-VHH had binding for all *Aspergillus* species and had no cross-reactivity with non-*Aspergillus* species.

Moreover, a nanobody-polyclonal antibody sandwich ELISA based on the PO8-VHH was optimized and developed. This assay was able to detect the fungus at a concentration as low as 1 μg mL^−1^, the same magnitude as reported by others^10^. It is a pity that the detection limit in peanut and maize was a fungal concentration of 2 μg mg^−1^, with less sensitivity than that reported (1 μg g^−1^) thus far^14^. Although the sensitivity is not as good as that of scFv^14^, but as others have reported^20, 21^, the nanobody is more stable than scFv and has so small molecular weight (15-kDa), and the most important advantage of nanobody is that it has strong and fast tissue penetration, which is able to enter the dense tissue such as solid tumors to play a role^27, 28^. As we have shown in Fig. 4, it is found that PO8-VHH could bind to the same antigen displayed a single band (45-kDa) in *A. flavus* and *A. parasiticus*, which is of great value for exploring target molecules in *Aspergillus* pathogens by using the advantage of penetrate the cell membrane. We will use the immunoaffinity column to enrich the single component and determine the target molecule by mass spectrometry in next studies. Of course, we will also be committed to developing a more sensitive method based on PO8-VHH, such as the construction of AP-labeled functional nanobodies, to improve the detection limit of *A. flavus* in future work.

When sandwich ELISA and the HPLC method were used to evaluate samples that were naturally contaminated with aflatoxins, *A. flavus* antigens were detected in all samples containing aflatoxins (Table 3). Therefore, the sandwich ELISA would have potential to detect aflatoxigenic *Aspergillus* in naturally contaminated samples. We found that there is still a certain regularity here: for example, samples GX73 and GX54 showed the lowest ELISA signal, and their aflatoxins were lowest; the ELISA signal of samples AF-inoculated, AP-inoculated and GX51 were top three, and their aflatoxins also ranked the top three. However, we also found that ELISA and aflatoxin levels determined by HPLC did not correspond totally in this study because there were instances when high levels of aflatoxin samples showed low ELISA readings in Table 3. This result may be due to the differences in substrate composition and growth conditions (temperature, pH, water activity) since they were collected from different sources that affect aflatoxin levels^29^.

In summary, we have developed a sandwich ELISA based on a nanobody and polyclonal antibody which is selective for effectively detecting aflatoxin producing strains of *Aspergillus* spp. in agricultural products. This method is likely to be useful for detecting the fungi before they are mature enough to produce high levels of aflatoxin. Thus this is an excellent early detection system. To our knowledge, this is the first example of the development of a nanobody for detection of *Aspergillus* pathogens.

## Materials and Methods

### Materials, culture media and buffers

A representative fungal strains *A. flavus* 3.4408 purchased from China Center for Microbial Culture Collection, is a wild-type, aflatoxin-producing strain. Other fungi (Table 2) were conserved in our laboratory. *Escherichia coli* ER2738 competent cells were purchased from Lucigen Corp. (Middleton, WI, USA), Top 10 F′ competent cells was obtained from Life Technologies (Grand Island, NY). Complete and incomplete Freund’s adjuvant, 3,3,5,5-tetramethylbenzidine (TMB), goat anti-mouse IgG antibody conjugated to horseradish peroxidase (HRP), and HRP-labeled goat anti-rabbit IgG antibody were purchased from Sigma Company (St. Louis, MO, USA). HRP-labeled mouse anti-M13 monoclonal antibody was purchased from GE Healthcare (Piscataway, NJ, USA). Anti-HA tag mouse monoclonal antibody was purchased from ComWin Biotech (Beijing, China). Helper phage M13KO7 and *Sfi*I were obtained from New England Biolabs (Ipswich, MA, USA). pComb3X phagemid vector was a generous gift from Dr. Carlos F. Barbas (The Scripps Research Institute, La Jolla, CA). LeukoLOCK total RNA isolation system was obtained from Applied Biosystems (Foster City, CA). QIAprep Spin MiniPrep Kit, QIAquick Gel Extraction Kit and QIAquick PCR Purification Kit were all from Qiagen. xTractor buffer for protein extraction and Ni-NTA chromatography resin were purchased from Clontech Laboratories, Inc. (Mountain View, CA, USA). The Costar 96-well EIA/RIA plate was purchased from Corning Incorporated (Corning, NY, USA).

Fungal culture: Czapek medium (3% (w/v) sucrose, 0.3% (w/v) NaNO_3_, 0.1% (w/v) K_2_HPO_4_, 0.05% (w/v) MgSO_4_·7H_2_O, 0.05% (w/v) KCl, 0.001% (w/v) FeSO_4_, pH6.5).

The following buffers were used: (1) 0.01 M phosphate buffered saline (PBS, pH 7.4) was prepared by adding 8 g of NaCl, 2.9 g of Na_2_HPO_4_·12H_2_O, 0.2 g of KH_2_PO_4_ and 0.2 g of KCl in 1000 mL deionized water, (2) coating buffer was 0.025 M, pH 9.6 carbonate buffer, (3) washing buffer was PBS containing 0.05% Tween 20 (v/v) (PBST), (4) block buffer was 5% skimmed milk (m/v) in PBST, (5) substrate solution system was composed by 9.5 mL pH 5.0 phosphate-citrate buffer, 0.5 mL 2 mg mL^−1^ TMB (dissolved by ethanol) and 32 μL 3% (w/v) urea hydrogen peroxide, (6) stop solution was 2 M H_2_SO_4_.

Unless otherwise stated, all other chemicals and organic solvents were of analytical reagent grade or better. Water was obtained from a MilliQ purification system (Millipore).

### Safety

This study involving the care and use of animals was carried out in strict accordance with the recommendations in the Guide for the Care and Use of Laboratory Animals of the National Institutes of Health. The protocol was approved by the Laboratory Animal Monitoring Committee of Hubei Province and performed accordingly.

Pure aflatoxin standards were handled in a hood with extreme caution. All items coming in contact with aflatoxins, phage and bacterial cultures (glassware, vials, tubes, ELISA plates, etc.) were immersed in a 10% bleach solution for 1–2 h before they were discarded or autoclaved.

### Antigen preparation and immunization

A representative isolate of *A. flavus* 3.4408, a potential aflatoxin producer was used for antigen preparation. The mycelia were cultured in Czapek medium on a shaker (200 rpm) at 28 °C for 5 days. The mycelia were harvested by filtration through four layers of cheesecloth. Extracellular antigens in the medium were prepared according to Tsai and Cousin^3^. In brief, the cultural fluid was separated by filtration and then through a 0.45-μm membrane. The filtrate was extracted with 80% saturated ammonium sulfate by stirring for 2 h at 4 °C. After the precipitate was suspended in PBS and then dialyzed overnight, the filtrate was concentrated and the concentration was determined. Intracellular antigens (mycelia lysate) prepared according to the method of Yong and Cousin^6^. For the preparation of mycelia lysate, mycelia were suspended in PBS with a final concentration of 3.7% formalin solution. After inactivation overnight at 4 °C, the mycelia were then frozen immediately in liquid nitrogen and grinded into fine powder. The powdered mycelia were weighed and suspended in PBS buffer. The suspended mycelia were homogenized for 3 times in a high pressure homogenizer ATS1500 at 100 bar and further up to 1000 bar for 4 times so as to fully disrupt mycelia. Disruptions of mycelia were confirmed by electron microscope.

A 2-year-old male alpaca was immunized subcutaneously with 1 mg mL^−1^ extracellular antigens and 100 mg mL^−1^ mycelia lysate (fungal concentration) mixed with the same volume of Freund’s complete Adjuvant. Five additional injections were given at 2-week intervals. After the last booster, 2 × 10 milliliters blood was used for mRNA extraction.

The New Zealand White female rabbit was injected intramuscularly with similar method of alpaca. Boosters were given every other week for a total of six times. Seven days after the final injection, the rabbit was bled to collect whole blood and antiserum. Immunoglobulin G (IgG) was purified from the antiserum using octanoic acid-saturated ammonium sulfate precipitation^30^. Then, the purified polyclonal antibody was freeze-dried and stored at −20 °C.

Preimmune serum was collected as a negative control. The antibody titers were assayed by indirect ELISA.

### Phage-displayed Library Construction

According to the titer results, the sixth blood was selected to extract RNA and construct the library. The library was constructed as previously described^24^. In brief, total RNA was extracted from alpaca’s blood and transcribed into cDNA. Nanobody gene fragments encoding the VHH variable domains were amplified by PCR using a previously published procedure^31^. Two pairs of primers containing two different *Sfi*I sites (underlined) are as followed, forward primer VHH-F (CAT GCC ATG ACT GTG GCC CAG GCG GCC CAG KTG CAG CTC GTG GAG TC) targeting the framework 1 region, reverse primers VHH-R1 (CAT GCC ATG ACT CGC GGC CGG CCT GGC CGT CTT GTG GTT TTG GTG TCT TGG G) and VHH-R2 (CAT GCC ATG ACT CGC GGC CGG CCT GGC CAT GGG GGT CTT CGC TGT GGT GCG) corresponding to the IgG2 and IgG3 hinge region, respectively. The PCR products and pComb3X phagemid vector were separately digested with *Sfi*I and subsequently ligated to generate pComb3X/nanobody constructs. The resulting recombinant phagemids were transformation into the competent cells of *E. coli* ER 2738. The cells were then plated on LB ampicillin agar plates to estimate the library size. Twenty clones were selected from the LB-amp plate and sequenced to evaluate library diversity.

### Panning

The constructed phage library containing approximately 10^8^ phage particles was screened against *A. flavus* through three rounds of panning. A solid phase protocol was performed to select the nanobodies with high affinity. Briefly, the wells were washed with PBST increased five times in each round (10, 15 and 20 times in the first, second and third rounds, respectively), while the amount of antigens were fixed. Under these stringent conditions, the ratios of output and input phages increased steadily (Table 1), with about 10-fold increased phage recovery after the third round of panning compared with the first one, demonstrating an efficient enrichment of specific antibodies. After three rounds of panning, 48 clones were randomly selected from the final output titer plate and infected with M13KO7 helper phage for further characterized by phage enzyme-linked immunosorbent assay (ELISA). Clones binding to antigens but not to any other protein like BSA or OVA were deemed positive and selected for further analyses.

### Sequencing analysis of nanobodies

The rescued phages were applied to phage ELISA with 1 mg mL^−1^ antigens as previously described^32^. Plasmid DNAs from the positive clones were extracted and sequenced using the primer gback (GCCCCCTTATTAGCGTTTGCCATC).

### Soluble nanobody ELISA

Soluble nanobodies were expressed in bacteria as previously described^26^.

The antigen-binding characteristics of nanobodies were identified by indirect ELISA. The protocol for indirect ELISA was similar to that described by Hu *et al*.^32^ with slight modification.

96-well microtiter plates were respectively coated with 100 μL of 100 μg mL^−1^ extracellular antigens and mycelia lysate from *A. flavus* and *A. parasiticus* in PBS per well overnight at 4 °C and blocking with 5% skimmed milk in PBS at 37 °C for 2 h. After three times washing with PBST, 200 nM purified nanobodies was added to each well and incubated for 1 h at 37 °C. After three washing cycles, 100 μL of anti-HA mouse monoclonal antibody was added to each well following by 1 h incubation at 37 °C. Then the plate wells were incubated with 100 μL of HRP-labeled goat anti-mouse IgG antibody for 1 h at 37 °C. After washing, 100 μL peroxidase substrate solution system were incubated for 15 min at 37 °C. Enzyme reactivity was stopped by adding 50 μL of 2 M H_2_SO_4_ and the absorbance was detected at 450 nm by a microplate reader. Some other protein like BSA or OVA served as the control.

To further evaluate the specificity of the nanobody, ten isolates of *Aspergillus* spp. with different level aflatoxin and other genera of fungi or bacteria were used for indirect ELISA analysis. The remaining steps were performed the same as soluble nanobody-ELISA.

Sensitivities of nanobodies were measured by an indirect ELISA. Different concentrations of *A. flavus* and *A. parasiticus* grinded in PBS (10^−2^, 10^−1^, 1, 10, 10^2^, 10^3^, 10^4^ μg mL^−1^) were added into plate wells and detected with 200 nM nanobodies in indirect ELISA.

### Immunoblot analysis of nanobody

Immunoblots of extracellular antigens and mycelia from *A. flavus* and *A. parasiticus* was detected with nanobodies. In immunoblot assays, extracellular antigens and mycelia of *A. flavus* and *A. parasiticus* boiled for 5 min were applied to 12% (w/v) SDS–PAGE and then transferred onto nitrocellulose membrane (Millipore). The membrane was washed with TBST three times for 15 min and then blocked with 5% non-fat milk for 1 h at room temperature, followed by incubation with nanobodies (100 nM) for 2 h. After incubating with the nanobody solution and washed with TBST, the membranes were followed by addition of anti-HA mouse antibody (1:1000 dilution) and HRP-labeled goat anti-mouse IgG antibody (1:2000 dilution) for 1 h at room temperature, respectively.

The membrane was then washed and incubated with ECL (USA) solution in a dark room for 10 min.

### Detection of *A. flavus* by sandwich ELISA

A sandwich ELISA procedure requires testing match pair antibodies, capture and detection antibodies that detect different epitopes on the target antigen so that they do not interfere with the other antibody binding. To optimize sandwich ELISA match pair antibodies through the nanobody and polyclonal antibody, the capture antibody and detection antibody were compared for their applicability and sensitivity.

Different concentrations of mycelia from *A. flavus* and *A. parasiticus* ranging from approximately 10^–2^ to 10^4^ μg mL^−1^ were determined by a sandwich ELISA according to the following protocol: 96-well microtiter plates were coated with 100 μL of capture antibody (nanobody) at 0.5 μg mL^−1^ at 4 °C overnight; three times washing with PBST were performed and each well was blocked with 300 μL of 5% non-fat milk at 37 °C for 2 h; after three times washing with PBST, 100 μL of ten-fold serial diluted mycelia (*A. flavus* and *A. parasiticus*) were added at 37 °C for 1 h; washing cycles were repeated and 100 μL of the polyclonal antibody at 1 μg mL^−1^ was added and incubated at 37 °C for 1 h; after washing, 100 μL HRP-labeled goat anti-rabbit IgG antibody (1:10000 dilution) was incubated at 37 °C for 1 h; after six times washing, 100 μL of TMB solution were incubated at 37 °C for 15 min; the reaction was then terminated by adding 50 μL of 2 M H_2_SO_4_ and the absorbance values was detected at 450 nm.

### Preparation of contaminated agricultural products samples for sandwich ELISA analysis

To determine the limit of quantification, 0.1 g of grinded peanut or maize suspended in 10 mL PBST was then 10-fold diluted and mixed with different concentrations of mycelia ranging from 10^−2^ to 10^4^ μg mg^−1^, a 100 μL homogenates was added into plate wells for sandwich ELISA analysis.

For analysis of the feasibility of nanobodies combined with polyclonal antibody for detecting *Aspergillus*-infected sample, sandwich ELISA was used to detect naturally infected peanut and maize samples collected from different areas and 4 samples inoculated with *A. flavus* and *A. parasiticus*, respectively (Table 3).

### Determination of aflatoxins in agricultural products samples

The method was described by Chen *et al*.^33^. Briefly, aflatoxins in peanuts or maizes were extracted and cleaned up using the AOAC method 991.31^34^. Aflatoxin immunoaffinity columns (IACs), with column capacity of 2000 ng, were made, utilising the antibody-amino silica gel microparticle^35, 36^. Quantitative analysis of aflatoxins was performed by Agilent 1220 HPLC, equipped with a fluorescence detector (FLD). A C18 analytical column (15 cm × 4.6 mm × 5 μm) and Romer Derivatisation Unit were used in the system.
